# Surface-modified FeCo nanoparticles: magnetic dilution and structure–magnetism relationships

**DOI:** 10.1039/d6ra04045d

**Published:** 2026-07-06

**Authors:** Bui The Huy, Jong Won Chung, The-Long Phan, Tran Thi Ngoc Nha, Le Thi Tuyet Ngan, Pham Thanh Phong

**Affiliations:** a Major of Biomedical Engineering, Division of Smart Healthcare, College of Information Technology and Convergence, Pukyong National University Busan 48513 Republic of Korea; b R&D Center, Vmaker Co. Daegu 41756 Republic of Korea; c Faculty of Physics Engineering and Nanotechnology, VNU University of Engineering and Technology, Vietnam National University 144 Xuan Thuy Road, Cau Giay Hanoi 100000 Vietnam; d Graduate University of Science and Technology, Vietnam Academy of Science and Technology 18-Hoang Quoc Viet Hanoi City Vietnam; e Faculty of Engineering and Technology, Thai Nguyen University of Information and Communication Technology, Thai Nguyen University Thai Nguyen City Vietnam; f Laboratory of Magnetism and Magnetic Materials, Science and Technology Advanced Institute, Van Lang University Ho Chi Minh City Vietnam phamthanhphong@vlu.edu.vn; g Faculty of Applied Technology, Van Lang School of Technology, Van Lang University Ho Chi Minh City Vietnam

## Abstract

FeCo nanoparticles with controlled composition and tailored surface chemistry were synthesized *via* an ultrasound-assisted co-precipitation method and subsequently functionalized with zirconium oxide (ZrO_2_), polyethylene glycol, and chitosan using precipitation, adsorption, and surface coupling strategies, respectively. Structural and morphological analyses by X-ray diffraction and electron microscopy confirm the formation of crystalline body-centered cubic (bcc) FeCo nanoparticles with preserved core structure after surface modification, while energy-dispersive X-ray spectroscopy verifies the successful incorporation of constituent elements without detectable impurity phases. Magnetic measurements show that pristine FeCo nanoparticles exhibit a high saturation magnetization (*M*_s_) of ∼187 emu g^−1^ with typical soft magnetic behavior at room temperature. After surface modification, all samples retain their intrinsic ferromagnetic characteristics; however, a systematic reduction in *M*_s_ is observed. This decrease is attributed to magnetic dilution from non-magnetic shells together with interfacial spin disorder at the FeCo surface. Among the investigated coatings, ZrO_2_ induces the strongest reduction in saturation magnetization, consistent with its higher density and more effective surface passivation. This effect is quantitatively described by an effective magnetically inactive thickness (*δ*_mag_), which accounts for both the physical coating layer and interfacial magnetic suppression. A nearly linear dependence between *M*_s_ and *δ*_mag_ is observed, suggesting that magnetic dilution is governed by a progressively growing interfacial inactive region rather than abrupt phase changes. These results demonstrate that surface engineering provides a robust route to tune the balance between magnetic performance and interfacial stability in FeCo nanoparticles without altering their fundamental magnetic nature. The combination of high magnetization, structural robustness, and controllable surface chemistry highlights their potential for advanced magnetic and bio-related applications.

## Abstract 2

FeCo nanoparticles with controlled composition were synthesized *via* ultrasound-assisted coprecipitation and functionalized with ZrO_2_, polyethylene glycol, and chitosan using precipitation, adsorption, and coupling strategies.

## Graphical abstract

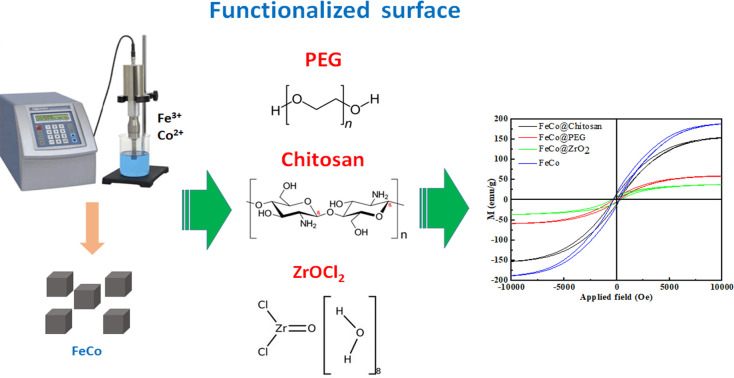

## 1 Introduction

Magnetic nanoparticles have attracted sustained and growing attention over the past two decades due to their unique size-dependent magnetic properties and their broad range of applications in biomedicine, catalysis, sensing, and data storage.^1–5^ In particular, colloidal magnetic nanoparticles have demonstrated significant potential in biomedical fields such as targeted drug delivery, magnetic hyperthermia,^4^ and magnetic resonance imaging (MRI),^5^ owing to their ability to respond to external magnetic fields while remaining superparamagnetic at physiological temperatures.

Among various magnetic materials, iron-based nanoparticles are the most extensively studied because of their relatively low toxicity and good biocompatibility.^4^ However, their saturation magnetization is generally limited. FeCo alloys represent an attractive alternative, as they exhibit exceptionally high saturation magnetization and magnetic permeability, making them promising candidates for applications requiring strong magnetic response at small particle sizes.^6–8^ Despite these advantages, the practical use of FeCo nanoparticles, particularly in biomedical applications, has been hindered by their poor chemical stability and susceptibility to oxidation, as well as concerns regarding cobalt-related toxicity.

Considerable efforts have therefore been devoted to the synthesis of FeCo nanoparticles with controlled size, narrow size distribution, and improved stability. Various synthetic approaches, including co-precipitation,^9,10^ hydrothermal synthesis,^11^ thermal decomposition,^12^ and chemical vapor condensation,^13^ have been reported. Among these methods, chemical co-precipitation from solution remains particularly attractive due to its simplicity, scalability, and feasibility at relatively low temperatures. Nevertheless, FeCo nanoparticles produced by wet-chemical routes are highly prone to surface oxidation, which can significantly degrade their magnetic performance.

Surface modification and encapsulation strategies have proven to be effective in enhancing the chemical stability and biocompatibility of magnetic nanoparticles. Organic and inorganic coatings not only protect the magnetic core from oxidation but also suppress particle aggregation and enable further functionalization. Commonly investigated coating materials include polymers such as polyethylene glycol (PEG) and chitosan,^14,15^ as well as inorganic shells such as silica SiO_2_, Al_2_O_3_, and metal oxides.^16,17^

While extensive studies have focused on coated iron oxide nanoparticles, reports on surface-modified FeCo nanoparticles remain relatively limited. Various surface-engineering strategies have been reported to improve the chemical stability, dispersibility, and functionality of FeCo and related magnetic nanoparticles. For instance, the FeCo coated with a graphite shell could prevent their degradation and isolate the particles from each other to avoid low-proximity interaction.^10,17^ Srinivacan's group^18^ used organic silica (3-aminopropyl)triethoxysilane (APTES) as a shell of FeCo. In addition, biocompatible coating materials such as PEG,^18^ chitosan, and zirconium oxide (ZrO_2_)^19,20^ have been widely employed in pharmaceutical and biomedical applications due to their low toxicity, chemical stability, and favorable interfacial properties.

To fabricate these nanostructures, ultrasound-assisted synthesis has emerged as a powerful approach, offering advantages such as enhanced mass transfer, reduced reaction temperature, and improved control over particle size and morphology. In another approach, the magnetization of air-stable FeCo, synthesized *via* the polyol method using polyethylene glycol, can reach about 200 emu g^−1^ at room temperature.^21^ However, this polyol method required the calcination of FeCo at 600 °C, and the obtained particle sizes were relatively large (∼90 nm).

Generally, while these inorganic oxide and polymeric shells provide oxidation resistance and enhanced thermal stability, surface modification often leads to changes in magnetic behavior. This degradation is typically due to the introduction of non-magnetic shell materials and interfacial spin disorder effects. Therefore, understanding the relationship between coating strategy and magnetic properties remains important for the design of FeCo-based core–shell nanomaterials.

In this work, we report an updated and efficient approach for the synthesis of FeCo nanoparticles using an ultrasound-assisted co-precipitation method, followed by surface modification with selected biocompatible coatings, namely polyethylene glycol, chitosan, and zirconium oxide. The effects of these coatings on the morphology, structural characteristics, and magnetic properties of the resulting nanoparticles are systematically investigated, with the aim of improving their stability and suitability for potential biomedical applications.

## 2 Experimental section

### 2.1 Materials

Iron(iii) chloride hexahydrate (FeCl_3_·6H_2_O), cobalt(ii) chloride hexahydrate (CoCl_2_·6H_2_O), zirconyl chloride octahydrate (ZrOCl_2_·8H_2_O, 98%), 3-aminopropyltriethoxysilane (APTES), sodium hydroxide (NaOH), acetic acid, monochloroacetic acid (MCA), poly(ethylene glycol) (PEG, *M*_n_ = 20 000), chitosan (MW > 300 kDa), *N*-(3-dimethylaminopropyl)-*N*′-ethylcarbodiimide hydrochloride (EDC·HCl), *N*-hydroxysuccinimide (NHS), ethanol (EtOH), isopropanol, and phosphate buffer solution (PBS) were of analytical grade and used as received without further purification. Deionized (DI) water was used throughout all experiments.

### 2.2 Synthesis of FeCo nanoparticles

FeCo nanoparticles were synthesized *via* an ultrasound-assisted co-precipitation method.^22^ In a typical procedure, FeCl_3_·6H_2_O and CoCl_2_·6H_2_O were dissolved in 50 mL of deionized water with a total metal ion concentration of 30 mM and a molar ratio [Fe^3+^]/[Co^2+^] = 1. The solution was heated to 70 °C under a nitrogen atmosphere and subjected to ultrasonic irradiation. Subsequently, a mixed solution containing 0.4 mol of hydrazine monohydrate and 0.06 mol of NaOH was slowly added. The reaction was maintained under ultrasonic conditions for a predetermined time. After completion, the resulting black precipitate was magnetically separated, washed repeatedly with deionized water until neutral pH was reached, and dried under vacuum at 40 °C for 10 h.

To ensure clarity in nomenclature, the notation FeCo@X (X = ZrO_2_, PEG and chitosan) is used throughout this work to denote surface-modified FeCo nanoparticles. The “@” symbol is employed as a convenient shorthand for surface functionalization and does not imply a fully continuous core–shell structure.

### 2.3 Surface modification with ZrO_2_

The surface modification of FeCo nanoparticles with ZrO_2_ was carried out according to the procedure reported in ref. 23 with some modifications. To introduce anchoring sites for zirconium species, the FeCo nanoparticles were first surface-functionalized with amino groups. Typically, 50 mg of FeCo nanoparticles were dispersed in a mixed solvent of ethanol (40 mL) and deionized water (5 mL) by ultrasonication for 30 min. Subsequently, 0.5 mL of APTES and 0.1 mL of acetic acid were added to the suspension, and the mixture was stirred at room temperature for 2 h. The resulting FeCo–APTES particles were collected by centrifugation, washed three times with ethanol to remove excess silane, and dried at 60 °C under vacuum overnight.

ZrO_2_ was then introduced onto the surface of the functionalized FeCo nanoparticles *via* a controlled heterogeneous precipitation process. In a typical procedure, 0.3 mmol of ZrOCl_2_·8H_2_O was dissolved in 20 mL of an ethanol/water mixture (5 : 3 v/v) and heated to 60–70 °C under continuous stirring. The FeCo–APTES particles were then added to the solution and stirred for 1 h to promote adsorption of Zr^4+^ species onto the functionalized nanoparticle surface. Subsequently, a dilute NaOH solution (0.1–0.2 M) was added dropwise using a syringe pump at a controlled rate of approximately 0.3–0.5 mL min^−1^ until the pH reached 7.5–8.0. The reaction mixture was maintained at 70 °C and aged for an additional 1–2 h to facilitate the formation of a Zr(OH)_4_ layer on the surface of the FeCo nanoparticles. The precipitate was collected by centrifugation, gently washed with deionized water, and dried at 60 °C under vacuum for 10–12 h. The dried FeCo–Zr(OH)_4_ precursor was subsequently converted into ZrO_2_-modified FeCo nanoparticles by stepwise calcination. The sample was heated to 300 °C and held for 1 h to remove residual solvent and chloride species, followed by heating to 450 °C for 1 h to promote Zr–O–Zr network formation. Finally, the temperature was increased to 600 °C and maintained for 1–2 h to achieve crystallization of the ZrO_2_ phase associated with the nanoparticle surface. Calcination was carried out under a nitrogen atmosphere to minimize oxidation of the FeCo nanoparticles.

### 2.4 Surface modification with polyethylene glycol

PEG-modified FeCo nanoparticles (FeCo@PEG) were prepared following ref. 24 with slight modifications. The PEG modification was performed *via* a sonochemically assisted surface adsorption process. Typically, 50 mg of FeCo nanoparticles were dispersed in 4 mL of ethanol by ultrasonication for 20–30 min. Separately, 10 mL of aqueous PEG solution (10–20 wt%) was prepared under vigorous stirring. The FeCo suspension was added dropwise into the PEG solution under continuous stirring, followed by sonication at 70 °C for 6–12 h to promote adsorption of PEG chains onto the nanoparticle surface *via* hydrogen bonding and weak coordination interactions. After reaction completion, the mixture was cooled to room temperature.

The PEG-modified nanoparticles were collected by magnetic separation, washed with ethanol and deionized water to remove excess PEG, and dried under vacuum at 50–60 °C overnight.

### 2.5 Surface modification with chitosan

Chitosan-functionalized FeCo nanoparticles (FeCo@chitosan) were synthesized *via* a covalent attachment strategy based on carbodiimide chemistry, adapted from ref. 25. Carboxymethyl chitosan (CMCS) was first prepared *via* alkaline carboxymethylation. Briefly, chitosan (3 g) and NaOH (15 g) were added to 100 mL of isopropanol/water mixture (80 : 20 v/v) and stirred at 60 °C for 30 min. Subsequently, monochloroacetic acid (20 mL, 7 M) was added dropwise, and the reaction was maintained at 60 °C for 4 h. The product was precipitated with ethanol, washed thoroughly, and dried at 50 °C under vacuum overnight.

For surface functionalization, 50 mg of FeCo nanoparticles were dispersed in 20 mL of PBS (pH 6.0) by ultrasonication for 20–30 min. EDC·HCl (0.1–0.2 mmol) and NHS (0.1–0.2 mmol) were added to activate the carboxyl groups of CMCS. After 15–30 min, CMCS solution (3 mg mL^−1^) was introduced into the FeCo suspension. The mixture was gently stirred at room temperature for 4–6 h to allow covalent coupling between CMCS and surface hydroxyl/oxide groups on FeCo nanoparticles.

The resulting FeCo@Chi nanoparticles were collected by magnetic separation, washed thoroughly with PBS and deionized water, and dried under vacuum at 50–60 °C.

### 2.6 *In vitro* cytotoxicity assay (MTT assay)

The biocompatibility of the synthesized FeCo@ZrO_2_ nanoparticles was evaluated using the MTT (3-(4,5-dimethylthiazol-2-yl)-2,5-diphenyltetrazolium bromide) assay. HeLa cells were seeded in 96-well plates at a density of 1 × 10^4^ cells per well and incubated at 37 °C in a 5% CO_2_ atmosphere for 24 hours to allow for cell attachment.

Following incubation, the culture medium was replaced with fresh medium containing various concentrations of FeCo@ZrO_2_ nanoparticles (ranging from 0 to 500 µg mL^−1^). After 24 hours of exposure, the treated cells were carefully washed three times with PBS. This gentle washing step was performed to remove any non-internalized or adhered magnetic particles, thereby minimizing potential interference with optical density (OD) measurements while simultaneously reducing mechanical stress on the cell monolayer.

Subsequently, 20 µL of MTT solution (5 mg mL^−1^ in PBS) was added to each well, and the plates were incubated for an additional 4 hours. The resulting formazan crystals were dissolved by adding 150 µL of dimethyl sulfoxide (DMSO) to each well with gentle shaking for 10 minutes. The absorbance was measured at a wavelength of 570 nm using a microplate reader. The cell viability was calculated relative to the untreated control group (100% viability) using the following equation:Cell viability (%) = (*A*_sample_/*A*_control_) × 100

All experiments were performed in triplicate to ensure statistical significance (*n* = 3).

### 2.7 Characterization

The morphology and particle size of the nanoparticles were examined using transmission electron microscopy (TEM, JEOL JEM-2100F). X-ray diffraction (XRD) patterns were recorded on a PANalytical X'Pert Pro MPD diffractometer using Cu-K_α_ radiation (2*θ* = 20–80°). Magnetic properties were measured using a vibrating sample magnetometer (VSM) module of the VersalLab Physical system from Quantum design (USA) at room temperature under an applied field of ±10 kOe. Fourier transform infrared (FTIR) spectra were obtained using a Jasco FT/IR-6300 spectrometer with KBr pellet preparation.

## 3 Results and discussion

### 3.1 Ultrasonic-controlled formation and purification of FeCo alloy phases

To optimize the formation and structural stability of FeCo nanoparticles synthesized *via* ultrasonic-assisted reduction, two parameters were systematically investigated: (i) sonication time and (ii) Fe : Co atomic ratio. The effect of ultrasonic duration on phase evolution was first examined to determine the optimal condition for complete alloy formation, followed by compositional optimization under the selected sonication condition.

**Fig. 1:**
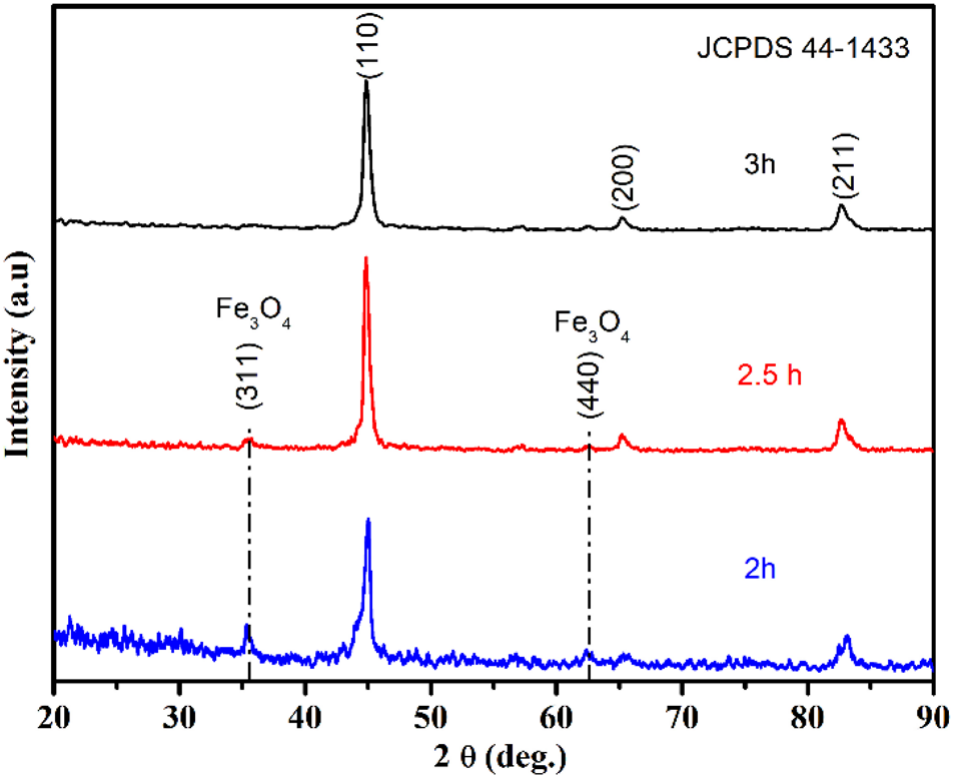
XRD patterns of FeCo for different sonicate time.

The X-ray diffraction (XRD) patterns of FeCo nanoparticles synthesized under different ultrasonic durations (2, 2.5, and 3 h) are shown in Fig. 1, aiming to identify the minimum sonication time required for complete alloy formation and phase purification. One can see that all samples exhibit three characteristic diffraction peaks at 2*θ* ≈ 44.82, 65.20, and 82.66°, corresponding to the (110), (200), and (211) diffraction planes of the body-centered cubic (bcc) FeCo structure, respectively. The observed reflections match well with the standard FeCo alloy phase (JCPDS no. 49-1433), confirming that the bcc FeCo lattice begins to form even at early stages of ultrasonic irradiation.

The lattice parameter calculated from the (211) reflection remains nearly constant at ∼2.85 Å for all samples, suggesting that the fundamental bcc lattice is established at the early stage of ultrasonic processing. This value is consistent with a partially ordered B2-type FeCo structure, reflecting efficient atomic intermixing between Fe and Co even at short sonication times. The average crystallite size, estimated by using the Scherrer equation from the (110) peak (with the shape factor *K* = 0.9), is approximately 12 nm for all samples, implying that the ultrasonic process primarily influences phase evolution rather than grain growth after nucleation.

However, phase purity strongly depends on sonication duration. At 2 and 2.5 h, additional reflections corresponding to Fe_3_O_4_ spinel phases are observed, indicating incomplete reduction and partial oxidation during early cavitation stages. With increasing sonication time, these impurity peaks gradually diminish and disappear completely at 3 h, resulting in a single-phase FeCo alloy. This evolution reflects the progressive enhancement of cavitation intensity with time, which increases localized temperature and pressure, thereby promoting both oxide reduction and Fe–Co interdiffusion. Based on these results, 3 h is identified as the optimal sonication time to ensure complete alloy formation and high structural purity, and this condition is used for subsequent compositional optimization.

**Fig. 2:**
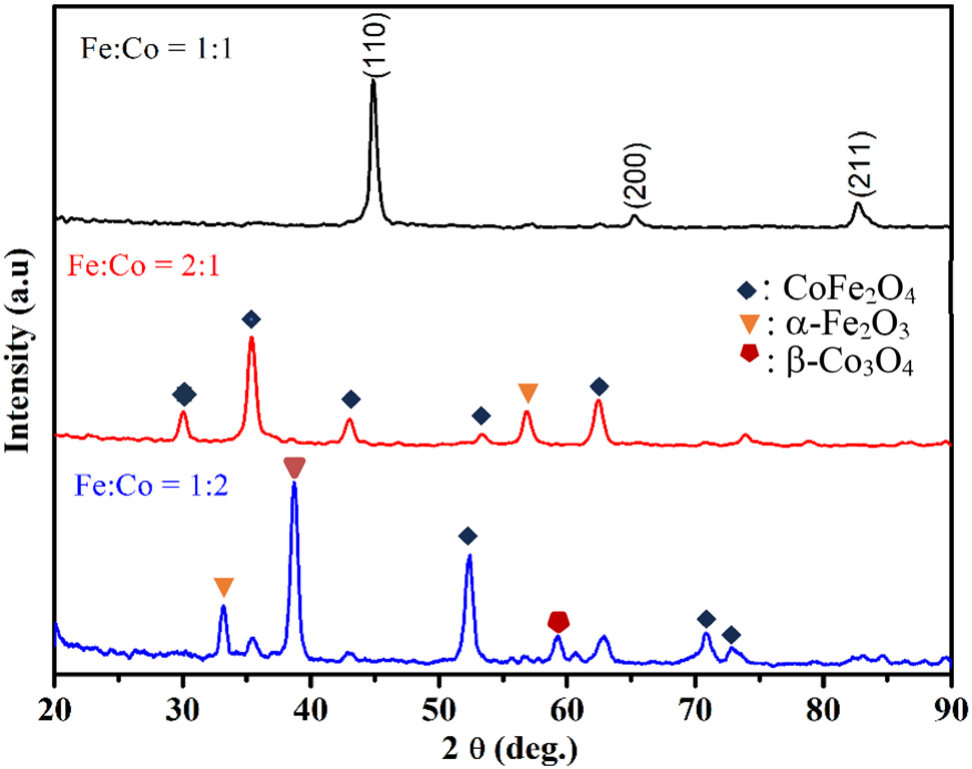
XRD patterns of FeCo for different ratios of Fe and Co.

The XRD patterns presented in Fig. 2 demonstrate the strong dependence of phase stability on the Fe : Co atomic ratio under the optimized sonication condition (3 h), allowing evaluation of the intrinsic thermodynamic stability of the FeCo alloy phase. For the equiatomic Fe : Co = 1 : 1 composition, all diffraction peaks can be indexed to the bcc FeCo structure, with no detectable secondary oxide phases. This indicates the formation of a chemically homogeneous alloy phase with high structural stability. The enhanced stability at this composition can be attributed to an optimal balance of Fe–Fe, Co–Co, and Fe–Co interactions, which promotes efficient atomic interdiffusion under cavitation-driven mixing. The structural characteristics further suggest short-range B2-type ordering within the bcc lattice.

The formation of the FeCo alloy phase was found to be strongly dependent on the Fe/Co precursor ratio. The equiatomic composition (Fe : Co = 1 : 1) favored the formation of a crystalline FeCo alloy, whereas non-equiatomic precursor ratios resulted in the appearance of oxide-related phases. This behavior can be attributed to differences in the reduction kinetics and oxygen affinity of Fe and Co species. Because Fe-containing intermediates are generally more susceptible to oxidation than Co species, any deviation from synchronized co-reduction conditions may promote the stabilization of oxide phases before complete alloy formation occurs. At the Fe : Co ratio of 1 : 1, simultaneous reduction and atomic interdiffusion between Fe and Co are more kinetically and thermodynamically favorable, thereby facilitating the formation of the alloy phase. Similar composition-dependent phase evolution has been widely reported for FeCo and other bimetallic magnetic nanoparticle systems.

In contrast, deviations from this equiatomic ratio (such as Fe : Co = 2 : 1 and 1 : 2) lead to a complete breakdown of the metallic alloy phase. Under these non-equiatomic conditions, the characteristic FeCo reflections disappear and are replaced by multiple oxide phases, including α-Fe_2_O_3_, Co_3_O_4_, and CoFe_2_O_4_ spinel structures. This phase transformation indicates that compositional imbalance destabilizes the metallic lattice, increasing the thermodynamic driving force for oxidation under sonochemical conditions. In Fe-rich and Co-rich systems, excess metallic species that cannot be incorporated into the alloy framework become preferentially oxidized due to their higher surface reactivity under cavitation-induced energetic environments. Consequently, the equiatomic Fe : Co = 1 : 1 composition represents a critical stability point where alloy formation is maximized and oxidation pathways are effectively suppressed, providing a robust structural foundation for the observed magnetic performance.

### 3.2 Composition-dependent structural and magnetic properties of FeCo nanoparticles

After optimizing the sonication condition, the effects of Fe : Co atomic ratio and thermal treatment on the structural and magnetic properties of FeCo nanoparticles were further investigated. The XRD patterns presented in Fig. 2 demonstrate the strong dependence of phase evolution on the Fe : Co atomic ratio after 3 h of ultrasonic treatment. For the equiatomic Fe : Co = 1 : 1 composition, all diffraction peaks can be indexed to the bcc FeCo alloy structure, with no detectable secondary oxide phases. This indicates the formation of a chemically homogeneous alloy phase with high structural stability under sonochemical conditions. The enhanced phase stability at this composition can be attributed to a near-optimal thermodynamic balance between Fe–Fe, Co–Co, and Fe–Co interactions, which promotes efficient atomic interdiffusion during cavitation-driven mixing. The observed structural characteristics also suggest a tendency toward short-range B2-type ordering within the bcc lattice, consistent with favorable electronic and atomic size compatibility between Fe and Co atoms. In contrast, deviations from the equiatomic ratio (Fe : Co = 2 : 1 and 1 : 2) result in a significant alteration of the phase constitution. The characteristic reflections of the FeCo alloy at (110), (200), and (211) are no longer observed. Instead, the diffraction patterns are dominated by multiple oxide phases, with peaks at 2*θ* ≈ 33.17, 38.17, 59.2, and 60.66° corresponding to iron oxides (α-Fe_2_O_3_ JCPDS no. 33-0664) and Co_3_O_4_ (JCPDS no. 42-1467) and cobalt-containing spinel phases such as CoFe_2_O_4_ (JCPDS no. 22-1086). This phase transition suggests that compositional imbalance disrupts the formation of a stable metallic lattice, thereby increasing the thermodynamic driving force for oxidation under sonochemical conditions. In Fe-rich and Co-rich systems, excess metallic species that are not incorporated into the alloy framework are more susceptible to oxidation due to enhanced surface reactivity and localized oxygen interaction during cavitation-induced high-energy events. Consequently, the equiatomic Fe : Co = 1 : 1 composition represents a critical stability point, where alloy formation is maximized and oxidation pathways are effectively suppressed. This optimized structural state provides a robust foundation for the superior magnetic properties observed in subsequent measurements.

**Fig. 3:**
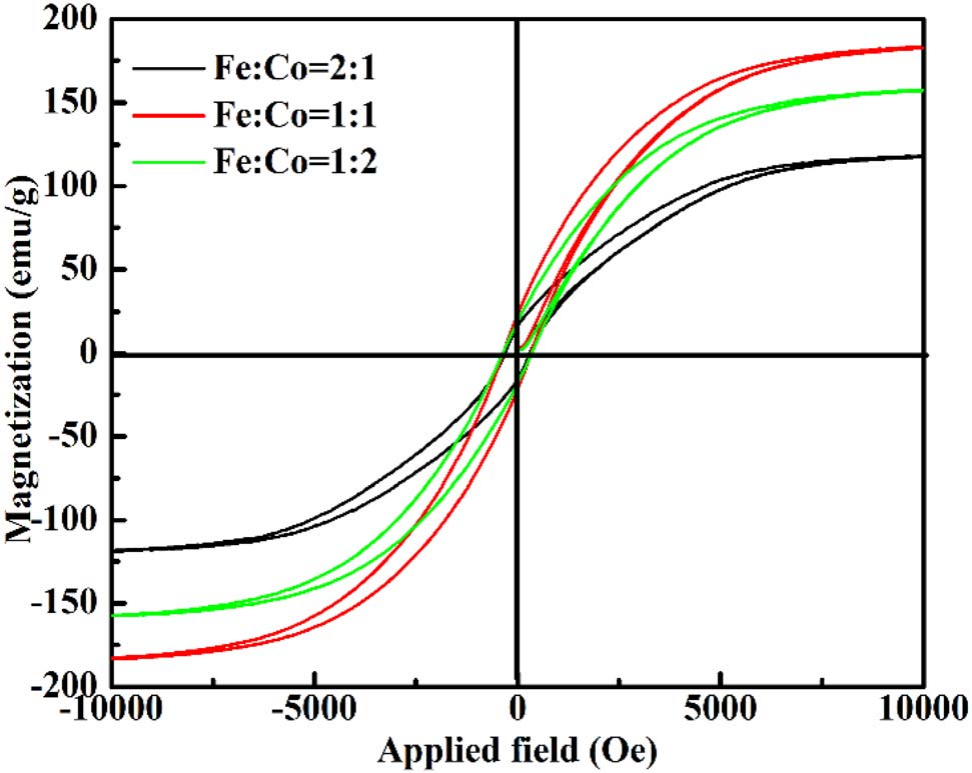
Magnetic behavior of three FeCo samples at room temperature with different ratios of Fe and Co.

Room-temperature magnetic hysteresis loops shown in Fig. 3 reveal a strong dependence of magnetic behavior on phase purity and atomic stoichiometry. The saturation magnetization (*M*_s_) values decrease systematically from 182 emu g^−1^ for the Fe : Co = 1 : 1 sample to 148 emu g^−1^ and 105 emu g^−1^ for the 1 : 2 and 2 : 1 compositions, respectively.

From the perspective of bulk alloy physics, this trend appears to contradict the prediction of the Slater–Pauling curve,^26^ where the magnetic moment of Fe–Co alloys is expected to reach a maximum near the Fe-rich composition (∼Fe_70_Co_30_) due to optimal 3d band filling. However, such predictions assume the preservation of a chemically homogeneous metallic alloy phase. In the present sonochemically synthesized nanoparticles, the magnetic response is governed primarily by phase stability rather than ideal electron concentration.

As XRD analysis demonstrated that only the equiatomic Fe : Co = 1 : 1 composition retained a single-phase metallic FeCo structure with a lattice parameter of approximately 2.85 Å. Under this condition, strong ferromagnetic exchange interactions between neighboring Fe and Co-3d orbitals are preserved, resulting in a high *M*_s_ approaching that of bulk FeCo alloys. The absence of secondary oxide phases further minimizes magnetic dilution and spin disorder, enabling efficient spin alignment throughout the nanostructure. In contrast, deviations from the equiatomic ratio lead to substantial phase transformation and corresponding changes in magnetic exchange mechanisms. For the Co-rich Fe : Co = 1 : 2 sample, the disappearance of the FeCo alloy reflections and the emergence of CoFe_2_O_4_-related phases indicate that the magnetic behavior is dominated by ferrimagnetic spinel interactions rather than metallic ferromagnetism. Although CoFe_2_O_4_ possesses relatively high magnetic anisotropy, its antiparallel sublattice spin configuration results in partial compensation of magnetic moments, thereby reducing the overall *M*_s_ compared with the metallic FeCo alloy. Additional spin canting and surface disorder at the nanoscale may further suppress the net magnetization.

A more pronounced reduction in *M*_s_ is observed for the Fe-rich Fe : Co = 2 : 1 sample, despite its nominal proximity to the Slater–Pauling maximum. Structural analysis indicates that this composition is dominated by hematite-related (α-Fe_2_O_3_) phases, which are weakly ferromagnetic or antiferromagnetic at room temperature. The formation of these oxide phases significantly suppresses long-range ferromagnetic exchange and acts as a magnetic dilution matrix, thereby reducing the overall magnetization. This result suggests that excess Fe atoms are highly susceptible to oxidation under sonochemical conditions, preventing the stabilization of the high-moment metallic FeCo phase.

Overall, the equiatomic Fe : Co = 1 : 1 composition represents the optimal balance between alloy stability and magnetic performance in the present sonochemical system. These findings demonstrate that, at the nanoscale, preservation of metallic phase purity is more critical than theoretical electron concentration alone in determining the magnetic properties of FeCo nanoparticles synthesized under oxidative environments.

**Fig. 4:**
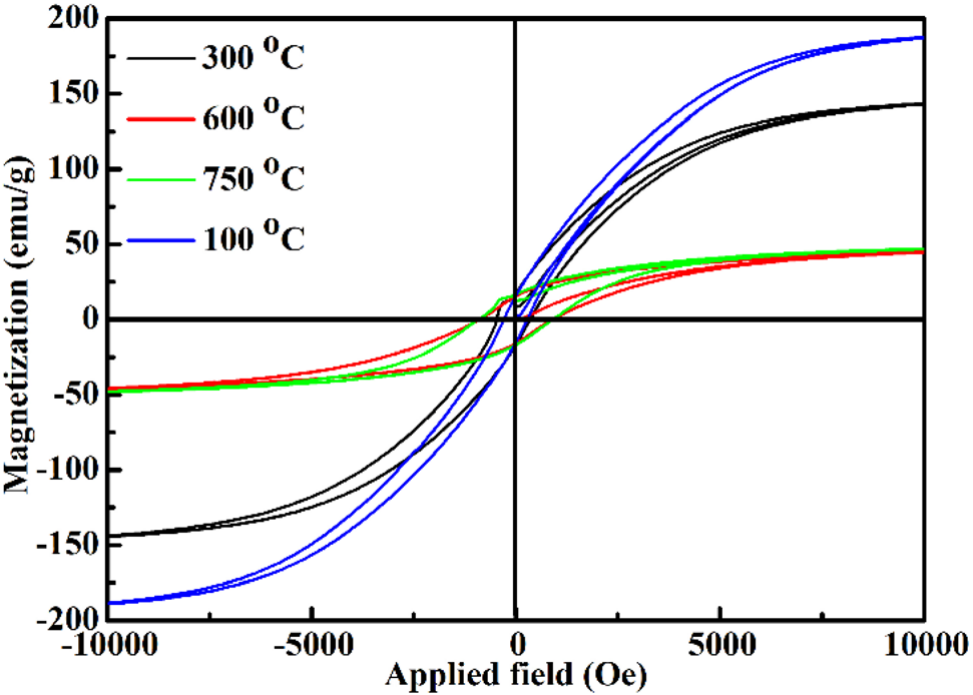
Magnetic behavior of FeCo samples at room temperature with different treatment temperature.

The FeCo nanoparticles with optimized Fe : Co = 1 : 1 composition were further subjected to thermal treatment at 100, 300, 600, and 750 °C to investigate the effect of post-synthesis annealing on their magnetic behavior. The corresponding room-temperature hysteresis loops are presented in Fig. 4. All samples exhibit typical ferromagnetic characteristics; however, both saturation magnetization (*M*_s_) and coercivity (*H*_c_) show a strong dependence on annealing temperature.

A slight increase in *M*_s_ is observed after annealing at 100 °C, where the magnetization reaches 187 emu g^−1^ compared to 182 emu g^−1^ for the as-prepared sample. This variation is small and lies close to the experimental uncertainty of VSM measurements; therefore, it should be interpreted as a weak but physically consistent change rather than a statistically significant enhancement. Such behavior can be associated with mild thermal relaxation effects, including partial removal of adsorbed species and reduction of surface spin disorder, which may slightly improve spin alignment without altering the crystal structure.

In contrast, further increase in annealing temperature leads to a pronounced reduction in saturation magnetization. The *M*_s_ decreases to 144 emu g^−1^ at 300 °C and further drops to approximately 45 emu g^−1^ at 600–750 °C. This strong decrease indicates a progressive loss of the high-moment metallic FeCo contribution. From a physical standpoint, such a drastic reduction is typically associated with thermally induced oxidation processes in Fe–Co systems, where the metallic phase becomes partially or fully transformed into lower-moment iron- and cobalt-containing oxides.

Although direct phase identification of the high-temperature annealed samples is not shown here, the observed magnetic evolution is fully consistent with the well-established behavior of FeCo nanoparticles reported in study of Yang *et al.*,^27^ where oxidation leads to the formation of ferrimagnetic or antiferromagnetic oxide phases with significantly reduced net magnetization compared to metallic FeCo. In particular, oxide phases such as iron oxides and cobalt ferrites are known to exhibit substantially lower saturation magnetization and higher magnetic anisotropy, which naturally explains both the reduction in *M*_s_ and the concurrent increase in coercivity.

Simultaneously, the coercivity (*H*_c_) increases monotonically with annealing temperature. This trend can be attributed to the progressive emergence of magnetically harder environments, including oxide-rich regions and increased structural disorder, which enhance magnetic anisotropy and domain wall pinning. Such effects are commonly observed in partially oxidized or nanostructurally evolved Fe–Co systems and do not require a specific phase assignment to be physically justified.

Overall, the M–H analysis demonstrates a clear transition in magnetic behavior induced by thermal treatment. Low-temperature annealing (100 °C) induces only weak perturbations associated with surface relaxation, whereas high-temperature treatment leads to a substantial degradation of magnetic performance, consistent with thermally driven oxidation and magnetic hardening effects. These findings highlight the importance of optimizing post-synthesis thermal conditions to preserve the high-moment metallic state of FeCo nanoparticles while maintaining desirable soft magnetic properties. Table 1 provides a comparison with previously reported studies.

**Table 1:** Comparison of *H*_c_, *M*_r_ and *M*_s_ values of FeCo nanoparticles with reported literature data

Size (nm)	*H*_c_ (Oe)	*M*_r_ (emu g^−1^)	*M*_s_ (emu g^−1^)	Ref.
12	220	15.8	187	This work
5000	104	NA	169	8
10–40	331	21	148	9
2.5	581.5	18	51	10
30	NA	NA	147.8	21
3000–5000	50	5	232	28
100	100	NA	200	29
450	215	NA	166	30
15	90	12.5	200	31
100	60	NA	142	32
165	117	10	211	33

### 3.3 Morphology, crystallinity, and surface functionalization of FeCo nanoparticles

**Fig. 5:**
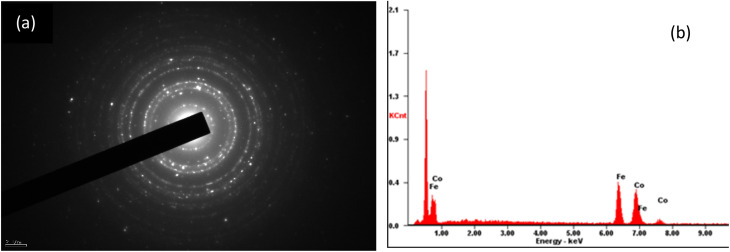
(a) SAED pattern of crystalline FeCo nanoparticles; (b) EDS spectrum confirming the Fe–Co elemental composition.

The selected-area electron diffraction (SAED) pattern of the FeCo nanoparticles annealed at 100 °C exhibits a series of well-defined concentric diffraction rings, confirming the polycrystalline nature of the nanostructures, as shown in Fig. 5a. The diffraction rings can be indexed to the (110), (200), and (211) crystallographic planes of the body-centered cubic (bcc) FeCo phase, with corresponding interplanar spacings of approximately 0.202, 0.143, and 0.117 nm, respectively. These values are consistent with standard bcc FeCo alloys and further support the structural observations obtained from XRD analysis.

Importantly, no additional diffraction rings associated with iron oxide or cobalt oxide phases are detected within the sensitivity of the SAED measurement, indicating that the nanoparticles predominantly retain their metallic FeCo structure after annealing at 100 °C. This result suggests that mild thermal treatment improves structural ordering without inducing significant oxidation, which is consistent with the enhanced saturation magnetization observed for this sample. The ring-like SAED image also indicates the presence of multiple randomly oriented nanocrystalline domains, characteristic of polycrystalline FeCo nanoparticles synthesized under sonochemical conditions. Such nanocrystalline metallic domains are favorable for maintaining strong ferromagnetic exchange interactions while preserving relatively soft magnetic behavior.

The elemental composition and chemical homogeneity of the nanoparticles were further examined by energy-dispersive X-ray spectroscopy (EDS), as shown in Fig. 5b. The EDS spectra collected from multiple regions reveal the presence of only Fe and Co signals, with an atomic ratio of approximately 51.7 : 48.3 at%, which closely matches the nominal precursor composition. This result confirms efficient alloy formation and homogeneous elemental distribution throughout the nanoparticles.

In addition, no significant oxygen signal is observed in the EDS spectra within the detection limit of the technique, suggesting that large-scale surface oxidation is limited under the present synthesis and low-temperature annealing conditions. Combined with the XRD and SAED analyses, these results demonstrate that the optimized synthesis route successfully stabilizes a structurally homogeneous FeCo metallic nanophase with minimal oxide contamination.

**Fig. 6:**
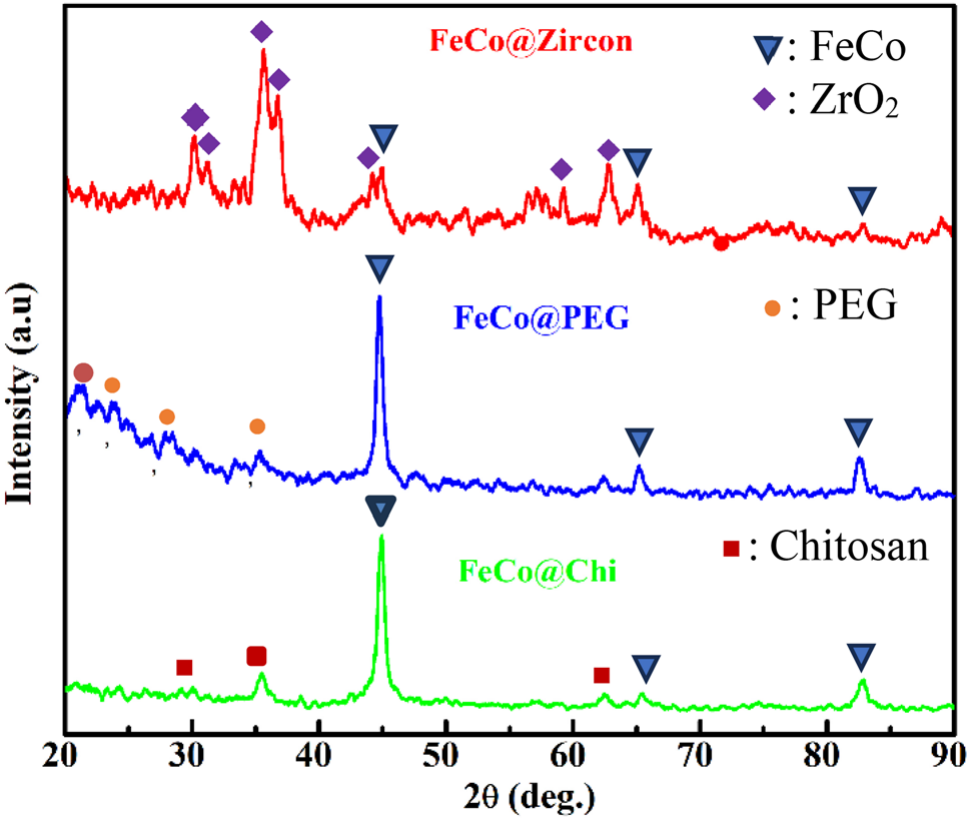
XRD patterns of FeCo different coating materials.

The XRD patterns of the coated FeCo nanoparticles, including FeCo@ZrO_2_, FeCo@PEG, and FeCo@chitosan, are presented in Fig. 6. For all surface-modified samples, the dominant diffraction peaks located at 2*θ* ≈ 44.8, 65.2, and 82.6° remain clearly visible and can be indexed to the (110), (200), and (211) planes of the bcc FeCo phase, respectively. The preservation of these characteristic reflections demonstrates that the crystalline structure of the FeCo core remains structurally stable after the coating process.

For the FeCo@PEG and FeCo@chitosan samples, no additional crystalline reflections associated with PEG or chitosan are detected. This behavior is expected because both materials are predominantly amorphous in nature and therefore contribute minimally to long-range diffraction signals. The absence of significant changes in the FeCo diffraction peak positions further suggests that the polymer coatings are formed primarily at the nanoparticle surface without altering the metallic core structure or inducing detectable phase transformation.

In contrast, the FeCo@ZrO_2_ sample exhibits several weak and broad reflections at lower and intermediate diffraction angles, which can be attributed to nanocrystalline or poorly crystalline zirconia-related phases. The broad nature and low intensity of these reflections indicate the formation of a thin surface oxide layer rather than bulk zirconia crystallization. Importantly, the characteristic FeCo peaks remain dominant and structurally unchanged, indicating that the ZrO_2_ shell acts mainly as a surface-protective layer while preserving the integrity of the metallic FeCo core.

Notably, no detectable diffraction peaks corresponding to iron or cobalt oxide impurities (such as Fe_3_O_4_, Fe_2_O_3_, or CoO) are observed in any of the coated samples within the sensitivity limit of the XRD measurement. This result suggests that the surface coatings help limit oxidation of the FeCo nanoparticles during post-synthesis processing and ambient exposure. In particular, the polymer shells (PEG and chitosan) likely provide steric and interfacial protection, whereas the ZrO_2_ layer functions as an inorganic diffusion barrier against oxygen penetration.

The preservation of the bcc FeCo phase after surface functionalization is highly significant for maintaining the superior magnetic properties of the metallic core while simultaneously introducing distinct surface chemistries for stability and potential biomedical compatibility. These observations confirm that the coating strategy successfully produces structurally stable FeCo-based core–shell nanoparticles without disrupting the underlying ferromagnetic alloy phase.

**Fig. 7:**
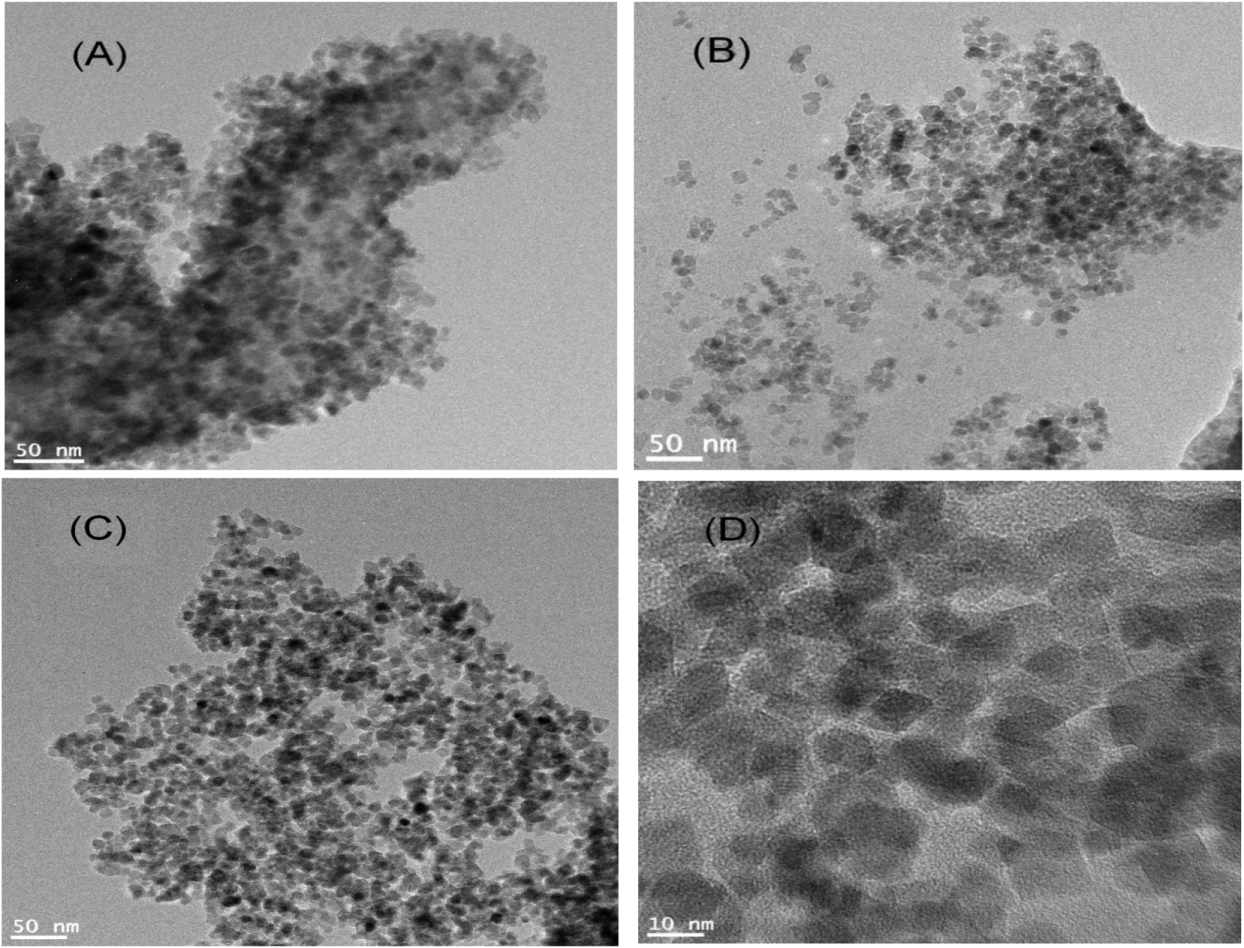
TEM images of (a) FeCo@chitosan, (b) FeCo@PEG, (c) FeCo@ZrO_2_, and (d) pristine FeCo nanoparticles.

The morphology and particle size of pristine and surface-modified FeCo nanoparticles were investigated by transmission electron microscopy (TEM), as shown in Fig. 7. Both uncoated and modified nanoparticles exhibit relatively uniform morphology with predominantly cubic-like nanostructures, characteristic of bcc FeCo nanoparticles synthesized under sonochemical conditions. The average particle size is estimated to be approximately 12–16 nm for all samples, indicating that neither the ultrasonic-assisted reduction process nor the subsequent surface-modification procedures significantly alter the dimensions of the FeCo nanoparticles. The relatively narrow particle-size distribution suggests that ultrasonic cavitation promotes rapid and homogeneous nucleation while suppressing excessive particle growth and agglomeration during synthesis. The preservation of nanoscale dimensions is particularly important for maintaining strong magnetic responsiveness and stable single-domain-like magnetic behavior. Furthermore, the absence of noticeable particle coarsening after surface treatment indicates that the modification processes do not induce significant structural degradation or particle coalescence.

The present TEM images do not provide unambiguous visualization of a continuous surface layer surrounding individual nanoparticles. This limitation arises from the combination of the small particle size, particle aggregation, and the limited contrast between the FeCo nanoparticles and the surface-bound inorganic or polymeric species. Therefore, the TEM observations alone cannot be considered direct evidence for the existence of a homogeneous coating layer.

Direct visualization of the surface-modified layer is challenging because of the limited contrast between the FeCo nanoparticles and the surrounding organic or ultrathin inorganic species in conventional TEM images. Therefore, evidence for surface modification is primarily obtained from complementary spectroscopic and compositional analyses. FT-IR spectroscopy reveals characteristic vibrational bands associated with PEG, chitosan, and zirconia-related species in the modified samples, while EDS confirms the presence of the corresponding elements. In addition, XRD analysis indicates that the crystalline bcc FeCo phase is preserved after surface treatment. Taken together, these results are consistent with successful modification of the FeCo nanoparticle surface without detectable alteration of the FeCo crystal structure.

**Fig. 8:**
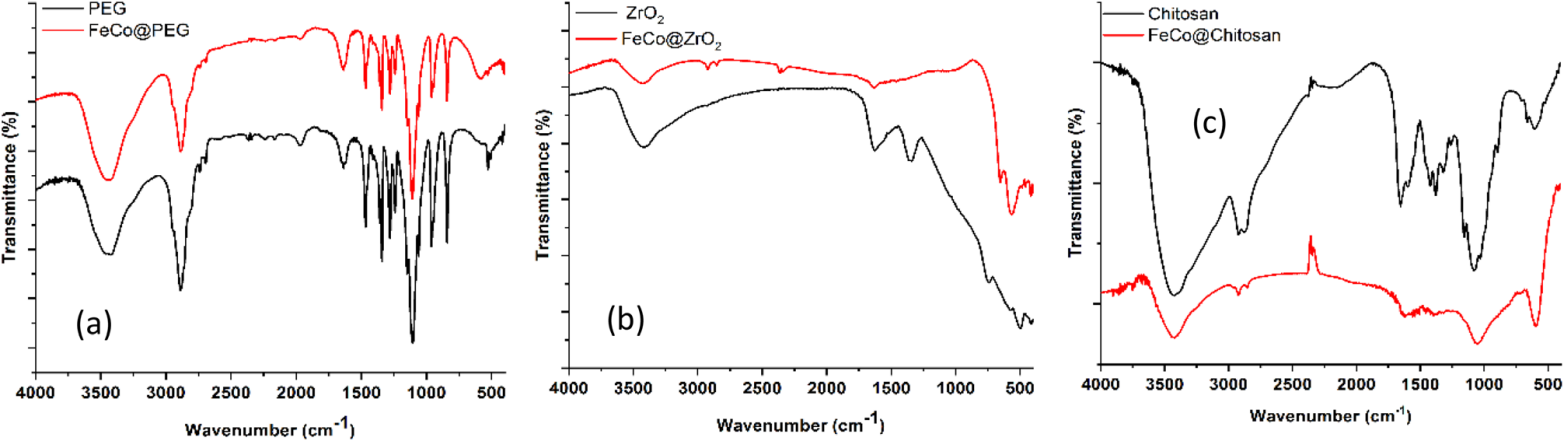
FT-IR of (a) pure PEG and PEG-coated FeCo; (b) ZrO_2_ and ZrO_2_-coated FeCo; (c) chitosan and chitosan coated FeCo.

The surface modification of FeCo nanoparticles with different shells was investigated by Fourier transform infrared (FT-IR) spectroscopy, as shown in Fig. 8. As illustrated in Fig. 8a, the FT-IR spectrum of pure PEG exhibits characteristic absorption bands at approximately 2890 cm^−1^, corresponding to the stretching vibration of –CH_2_ groups, 1095 cm^−1^ assigned to the C–O–C stretching vibration, and 958 cm^−1^ attributed to out-of-plane C–H bending. These characteristic PEG-related bands are also observed in the spectrum of PEG-coated FeCo nanoparticles, although with reduced intensity and slight broadening. This attenuation and band broadening suggest the successful surface functionalization of FeCo nanoparticles by PEG, likely through physical adsorption and interfacial interactions between PEG chains and the nanoparticle surface.

The FT-IR spectra of FeCo@ZrO_2_ nanoparticles are shown in Fig. 8b. For pure ZrO_2_, the absorption bands at approximately 740 cm^−1^ and 500 cm^−1^ are assigned to the asymmetric stretching vibration of Zr–O–Zr and the Zr–O stretching mode, respectively, confirming the formation of zirconia-related structural units.^34^ In the FeCo@ZrO_2_ sample, the band initially located at 740 cm^−1^ shifts to around 650 cm^−1^, which may reflect changes in the local bonding environment of zirconia species after deposition onto the FeCo nanoparticles. The presence of zirconia-related vibrational bands together with the observed spectral shift is consistent with the incorporation of zirconia species in the modified FeCo sample and suggests interaction between zirconia and the FeCo nanoparticle surface.

Fig. 8c presents the FT-IR spectra of pure chitosan and FeCo@chitosan nanoparticles. The absorption band around 604 cm^−1^ is attributed to Fe–Co lattice vibrations, confirming the presence of the metallic core. Compared with pure chitosan, the C–O stretching vibration shifts from 1089 cm^−1^ to 1083 cm^−1^ in the FeCo@chitosan spectrum, indicating possible coordination interactions between chitosan functional groups and the FeCo surface. In addition, the band at approximately 1602 cm^−1^ in pure chitosan, associated with N–H bending vibrations, shifts to around 1660 cm^−1^ after coating, suggesting a modification of the local chemical environment of amine groups upon interaction with the nanoparticle surface. The broad absorption band around 3425 cm^−1^ observed in all spectra corresponds to overlapping –OH and –NH stretching vibrations,^35,36^ further confirming the presence of chitosan on the nanoparticle surface.

Overall, the FT-IR results provide spectroscopic evidence for the presence of PEG-, chitosan-, and zirconia-related species in the modified samples. When considered together with the XRD, TEM, SAED, and EDS analyses, the data consistently indicate successful surface modification of FeCo nanoparticles while preserving the crystalline bcc FeCo phase and nanoscale morphology.

### 3.4 Magnetic properties of surface-modified FeCo nanoparticles: magnetic dilution and interfacial effects

**Fig. 9:**
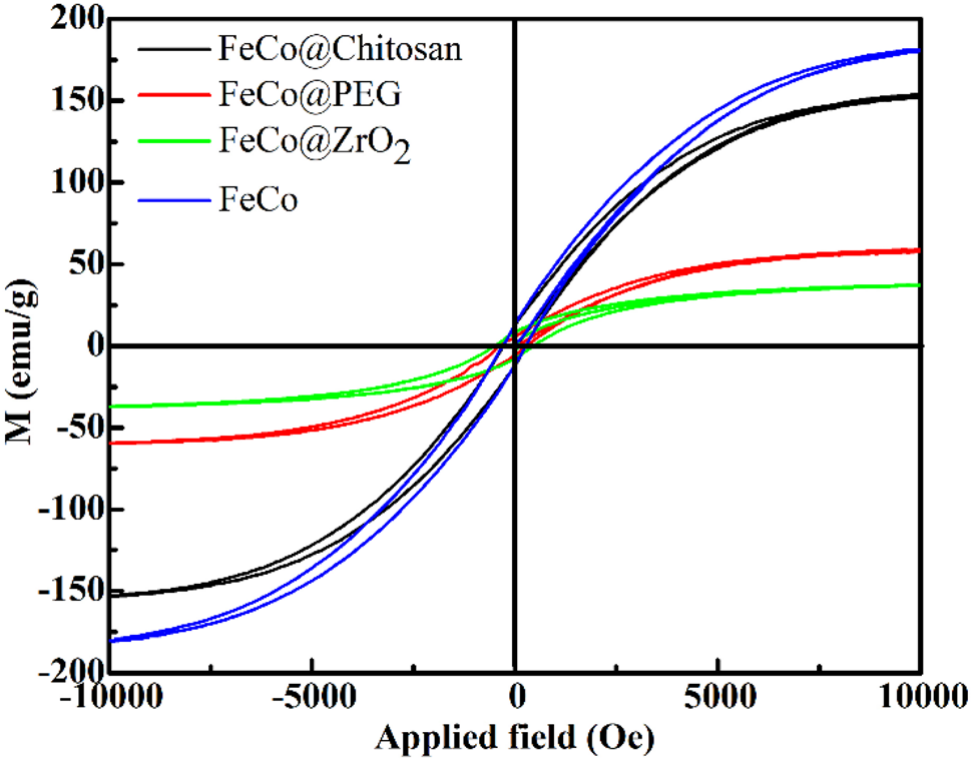
Magnetic behavior of FeCo samples with different surfactants.

Fig. 9 presents the room-temperature magnetic hysteresis loops of pristine FeCo nanoparticles and surface-modified samples, including PEG-, ZrO_2_-, and chitosan-treated FeCo nanoparticles. All samples exhibit typical S-shaped hysteresis curves with negligible coercivity, indicating that the soft magnetic character of the FeCo nanoparticles is preserved after surface modification. The absence of significant changes in loop shape suggests that the long-range ferromagnetic ordering of the FeCo core remains largely intact across all surface treatments.

Compared with pristine FeCo nanoparticles, all surface-modified samples show a reduction in saturation magnetization (*M*_s_). This reduction can be understood within a unified framework involving two main contributions: (i) magnetic dilution due to the presence of non-magnetic surface species when magnetization is normalized to total mass, and (ii) interfacial spin disorder and partial spin canting at the FeCo surface, which effectively reduces the contribution of surface atomic layers to the net magnetization. These effects are particularly relevant in nanoscale systems where surface-to-volume ratios are high.

A comparative analysis of different surface modifications reveals that the magnitude of *M*_s_ reduction depends not only on the amount of coating but also on the chemical nature and physical characteristics of the surface layer. For PEG- and chitosan-modified FeCo nanoparticles, the decrease in *M*_s_ is relatively moderate and can be primarily attributed to magnetic dilution caused by low-density polymeric layers. These organic coatings interact weakly with the metallic core and therefore introduce limited perturbation to interfacial spin alignment.

In contrast, ZrO_2_-modified FeCo nanoparticles exhibit the most pronounced decrease in saturation magnetization. This behavior is not solely a consequence of higher mass density, but also reflects stronger interfacial perturbation associated with the rigid inorganic nature of the ZrO_2_ layer. The formation of a chemically robust interface may enhance spin disorder and reduce exchange coupling at the FeCo surface, leading to a larger effective magnetically inactive region compared with polymer-coated samples.

Despite these differences in *M*_s_, all coated samples maintain similarly low coercivity, confirming that the fundamental soft magnetic behavior of the FeCo core is not significantly altered by surface modification. This indicates that the primary effect of surface treatment is the modulation of the effective magnetic moment rather than a change in intrinsic ferromagnetic exchange interactions.

Overall, the observed magnetic behavior can be consistently interpreted within a framework where the saturation magnetization is governed by the combined effects of magnetic dilution and surface-dependent interfacial spin disorder. Importantly, the results demonstrate that the magnetic response of FeCo nanoparticles is more strongly influenced by the chemical nature of the surface modification than by coating thickness alone. This highlights the role of interface engineering in tuning magnetic performance while preserving the intrinsic magnetic nature of FeCo nanoparticles.

To quantitatively correlate the magnetic response with surface modification, a simplified magnetic dilution framework is employed to describe the reduction in saturation magnetization of surface-modified FeCo nanoparticles. Within this approach, the measured saturation magnetization (*M*_sc_) is expressed in terms of the magnetic contribution of the FeCo core relative to the total mass of the nanoparticle system:1
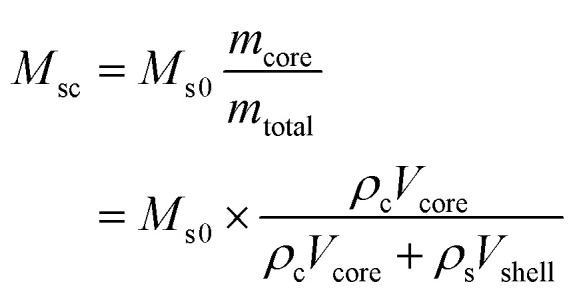
where *M*_s0_ is the saturation magnetization of the uncoated FeCo nanoparticles (187 emu g^−1^), while *ρ*_c_ and *ρ*_s_ denote the effective densities of the FeCo core and the surface-modified region, respectively.

This formulation is based on the assumption that the dominant contribution to the reduction in *M*_s_ arises from magnetic dilution due to the presence of non-magnetic surface species when magnetization is normalized to total sample mass. In addition to this geometric contribution, interfacial magnetic effects such as spin disorder, spin canting, and reduced exchange coupling at the FeCo surface are not explicitly separated in the model and are therefore incorporated into an effective magnetically inactive surface contribution.

The core and surface volumes are expressed as:2
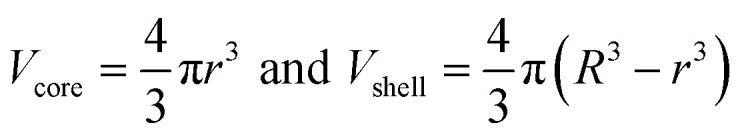


From eqn (1), the effective magnetic core radius (*r*) can be derived as:3
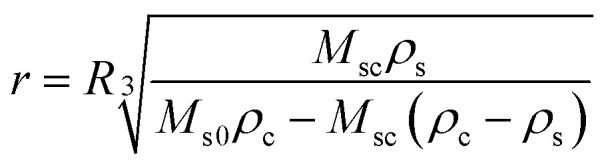


The corresponding effective magnetic shell thickness is defined as:

4 *δ*_mag_ = *R* − *r*

In this context, *δ*_mag_ should be interpreted as an effective parameter representing the total magnetically inactive region surrounding the FeCo nanoparticles. This region includes both the physically present surface layer introduced by PEG, chitosan, or ZrO_2_, and the interfacial zone where reduced exchange coupling and surface spin disorder suppress the net magnetic moment.

Although the model adopts a simplified spherical approximation, the FeCo nanoparticles exhibit a quasi-cubic morphology as observed in TEM analysis. This introduces a deviation from the ideal geometrical assumption. However, since all samples share comparable morphology and size distribution, the resulting systematic error is expected to be consistent across the dataset. Consequently, *δ*_mag_ should not be interpreted as an absolute geometrical thickness, but rather as a comparative descriptor of relative magnetic depletion among different surface chemistries.

In addition, interparticle dipolar interactions are not explicitly included in the model. This approximation is justified because saturation magnetization is extracted under high applied fields (±10 kOe), where magnetic moments are nearly fully aligned with the external field, significantly reducing the contribution of dipolar coupling to the measured *M*_s_.

Within this framework, the reduction in *M*_s_ is governed primarily by magnetic dilution and interfacial magnetic disorder rather than intrinsic modification of the FeCo core magnetization. The preservation of the bcc FeCo crystal structure observed in XRD and SAED supports the assumption that *M*_s0_ remains approximately constant across all samples.

Finally, comparison between *δ*_mag_ and the physical thickness estimated from TEM (*δ*_TEM_) provides additional insight into interfacial magnetic effects. For FeCo@chitosan, *δ*_mag_ is relatively close to *δ*_TEM_, suggesting limited additional magnetic perturbation beyond the physically observed surface layer. In contrast, FeCo@PEG and especially FeCo@ZrO_2_ exhibit larger *δ*_TEM_ values than *δ*_TEM_, indicating the presence of extended magnetically inactive regions arising from stronger interfacial spin disorder and reduced exchange coupling at the nanoparticle surface. The calculated *δ*_mag_ values and the physical shell thicknesses observed from TEM (*δ*_TEM_) are summarized in Table 2.

**Table 2:** Comparison between physical (TEM) and effective magnetic shell thickness for coated FeCo nanoparticles

Sample	*M*_s_ (emu g^−1^)	*ρ*_s_ (g cm^−3^)	*R*_TEM_ (nm)	*δ*_TEM_ a (nm)	*δ*_mag_ (nm)
FeCo@ZrO_2_	35	5.68	7.0	1.0	3.37
FeCo@chitosan	153	1.00	7.0	1.0	2.03
FeCo@PEG	58	1.20	8.0	2.0	4.84

a The shell thickness (*δ*_TEM_) was estimated from the difference between the average particle size after coating and the core size of uncoated FeCo nanoparticles, assuming a simplified core–shell geometry. This value should be regarded as an apparent geometrical thickness rather than a directly resolved TEM feature.

For the FeCo@ZrO_2_ sample, the substantial reduction in *M*_s_ suggests that partial oxidation and/or strong interfacial spin disorder may occur at the FeCo–ZrO_2_ interface during shell formation, resulting in the development of a magnetically dead layer. These findings indicate that although all coating agents successfully provide surface protection and functionalization, chitosan appears to be the most effective coating for preserving the intrinsic magnetic performance of FeCo nanoparticles.

**Fig. 10:**
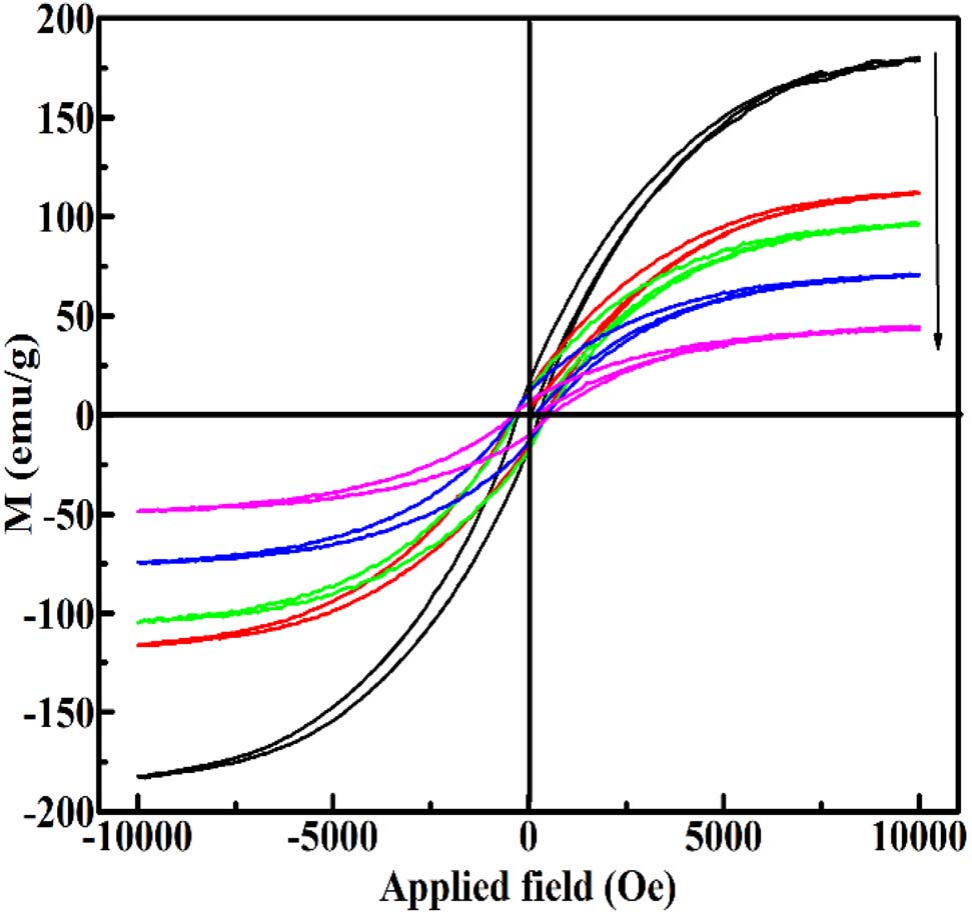
ZrO_2_ precursors concentration dependence of magnetization for FeCo@ZrO_2_.

Although all three coating materials -chitosan, PEG, and ZrO_2_ – lead to a reduction in saturation magnetization, the underlying coating mechanisms are fundamentally different. For polymeric shells such as PEG and chitosan, the coating thickness and mass fraction are governed mainly by adsorption and surface grafting processes, making quantitative control of shell thickness difficult using precursor concentration alone. In contrast, ZrO_2_ shells are formed through a controlled inorganic precipitation process, enabling systematic modulation of shell thickness by adjusting the amount of ZrOCl_2_·8H_2_O precursor. Consequently, the FeCo@ZrO_2_ system provides an appropriate platform for quantitatively investigating the relationship between shell growth and magnetic response, as illustrated in Fig. 10. The influence of ZrO_2_ precursor concentration on the magnetic properties of FeCo nanoparticles was evaluated by varying the ZrOCl_2_·8H_2_O precursor concentration while maintaining a constant FeCo core mass of 50 mg. As shown in the magnetic hysteresis curves (Fig. 10), *M*_s_ decreases systematically with increasing precursor concentration from 30 to 300 µmol. This behavior indicates a strong dependence of the magnetic response on the amount of non-magnetic ZrO_2_ incorporated into the core–shell structure.

The reduction in *M*_s_ originates primarily from the magnetic dilution effect associated with the increasing non-magnetic shell fraction. As more ZrO_2_ is deposited onto the FeCo surface, the relative mass contribution of the magnetic metallic core decreases when the magnetization is normalized to the total composite mass. Under the simplified assumption that the FeCo core largely retains its intrinsic magnetic moment, a thicker ZrO_2_ shell naturally results in a lower measured *M*_s_.

In addition to mass dilution, interfacial magnetic effects may also contribute to the observed decrease in magnetization. The formation of the ZrO_2_ shell can induce surface spin canting, interfacial exchange disruption, and the development of magnetically disordered regions at the FeCo–ZrO_2_ boundary. These effects reduce the effective magnetic contribution of surface Fe and Co atoms and become increasingly important as the shell thickness increases. Such interfacial magnetic suppression is particularly significant in nanoscale systems due to the high surface-to-volume ratio of the FeCo nanoparticles.

Despite the progressive decrease in *M*_s_, all coated samples retain similar S-shaped hysteresis loops with relatively low coercivity, indicating that the soft magnetic character of the FeCo core is largely preserved after shell formation. This observation suggests that the ZrO_2_ coating primarily modifies the magnitude of the magnetization rather than fundamentally altering the ferromagnetic nature of the FeCo nanoparticles.

The effective magnetic shell thickness (*δ*_mag_) of the FeCo@ZrO_2_ series was estimated using eqn (3) based on a simplified spherical core–shell model with homogeneous shell distribution. As the saturation magnetization decreases from 178 to 43 emu g^−1^, the calculated *δ*_mag_ increases from approximately 0.14 nm to 3.42 nm (Table 3), indicating progressive growth of the non-magnetic interfacial region surrounding the FeCo core.

**Table 3:** The effective magnetic shell thickness for ZrO_2_ – coated FeCo nanoparticles

*M*_s_ (emu g^−1^)	*M*_sc_/*M*_s0_ (emu g^−1^)	*r* (nm)	*δ*_mag_ (nm)
178	0.952	5.86	0.14
112	0.599	4.60	1.40
96	0.513	4.19	1.81
70	0.374	3.48	2.52
43	0.230	2.58	3.42

For the sample with *M*_s_ = 178 emu g^−1^, the extremely small *δ*_mag_ value (∼0.14 nm) suggests the formation of an ultrathin interfacial layer that provides initial surface passivation with minimal influence on the magnetic core. As the precursor concentration increases, the effective magnetic shell thickness expands progressively, indicating increased magnetic dilution and stronger interfacial magnetic suppression. For the sample with *M*_s_ = 43 emu g^−1^, the calculated *δ*_mag_ of 3.42 nm implies that a significant fraction of the nanoparticle volume no longer contributes effectively to the overall magnetization. Importantly, *δ*_mag_ should be interpreted as an effective magnetic thickness rather than the exact physical shell thickness, since it includes contributions from both the structural ZrO_2_ shell and magnetically disordered interfacial regions. The discrepancy between magnetic and physical thickness therefore reflects the degree of interfacial magnetic degradation occurring during shell growth.

Overall, these results demonstrate that the magnetic properties of FeCo@ZrO_2_ nanoparticles can be systematically tuned through controlled shell formation. Lower precursor concentrations preserve higher magnetic responsiveness, whereas thicker ZrO_2_ shells provide enhanced surface coverage and interfacial protection at the expense of saturation magnetization. This trade-off highlights the critical importance of optimizing shell thickness to balance magnetic performance and structural stability for targeted functional applications.

**Fig. 11:**
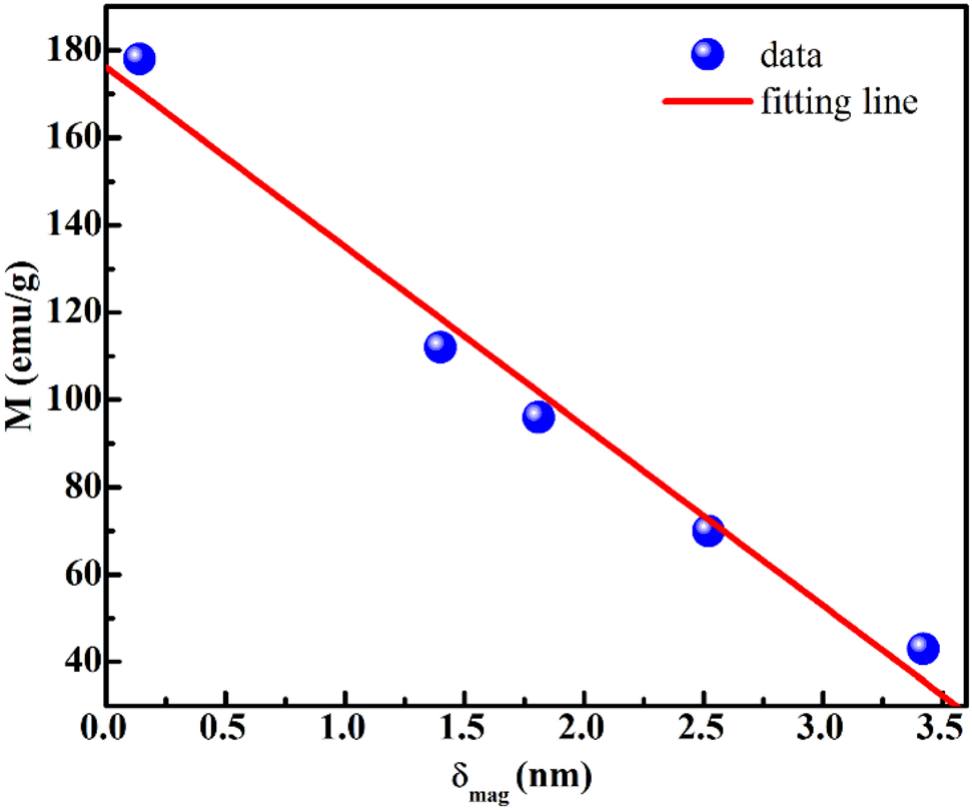
Dependence of saturation magnetization (*M*_s_) on the effective magnetic shell thickness (*δ*_mag_) for FeCo@ZrO_2_ nanoparticles.

Furthermore, as shown in Fig. 11, the saturation magnetization (*M*_s_) exhibits an approximately linear decrease with increasing effective magnetic shell thickness (*δ*_mag_). This trend indicates that the reduction in magnetic response is primarily governed by the progressive increase of the magnetically inactive surface region surrounding the FeCo core.

The near-linear dependence suggests that, within the investigated thickness range, each incremental increase in *δ*_mag_ contributes to a nearly proportional reduction in the effective magnetic volume fraction. This behavior is consistent with a gradual and uniform development of surface modification rather than abrupt structural transitions or heterogeneous phase formation.

From a physical standpoint, this linear-like scaling implies that the dominant contribution to the *M*_s_ reduction arises from geometric dilution effects combined with interfacial spin disorder, both of which scale proportionally with increasing surface-to-volume ratio. In contrast, no evidence of sudden deviations or saturation in the trend is observed, indicating that the intrinsic ferromagnetic core remains structurally stable across the coating range investigated.

It is important to note that the present model provides an effective description of the magnetic response based on a simplified core–shell approximation. In particular, the model does not explicitly account for nanoscale complexities such as spatially varying spin canting, local chemical inhomogeneity at the interface, or possible weak interparticle magnetic coupling.

Despite these simplifications, the consistency between the experimentally observed *M*_s_ trend and the model prediction suggests that the dominant magnetic response is well described by a combination of magnetic dilution and interfacial magnetic disorder. Therefore, the extracted *δ*_mag_ values should be interpreted as an effective parameter representing the total magnetically inactive region rather than a strictly geometrical shell thickness.

Importantly, because all samples were synthesized and measured under identical conditions, any systematic deviation from ideal behavior is expected to affect all data points in a similar manner. As a result, the relative trend of *M*_s_*versus δ*_mag_ remains robust and reliable for comparative analysis.

### 3.5 Cytocompatibility of FeCo@ZrO_2_ nanoparticles

The cytotoxicity of transition-metal nanoparticles is often associated with their ability to induce oxidative stress, disrupt cellular functions, or release potentially harmful metal ions into the biological environment. For this reason, surface modification is commonly employed to improve the chemical stability and biological compatibility of metallic nanoparticles intended for biomedical applications.^37–39^

Among the investigated surface-modified FeCo systems, cytocompatibility was evaluated specifically for the ZrO_2_-modified FeCo nanoparticles. The objective of this experiment was not to establish a comprehensive comparison among different coating chemistries, but rather to provide a proof-of-concept assessment of whether the introduction of a chemically stable inorganic surface layer can preserve acceptable biological compatibility while maintaining the desirable magnetic properties of FeCo nanoparticles. PEG and chitosan are already widely recognized as biocompatible surface modifiers for magnetic nanoparticles and, in the present work, were primarily employed to investigate surface functionalization and magnetic–property evolution. In contrast, ZrO_2_ was introduced mainly as a chemically inert protective layer designed to enhance the environmental stability of the FeCo surface. Therefore, FeCo@ZrO_2_ was selected as a representative system to evaluate whether such inorganic surface modification compromises the biological compatibility of FeCo nanoparticles.

**Fig. 12:**
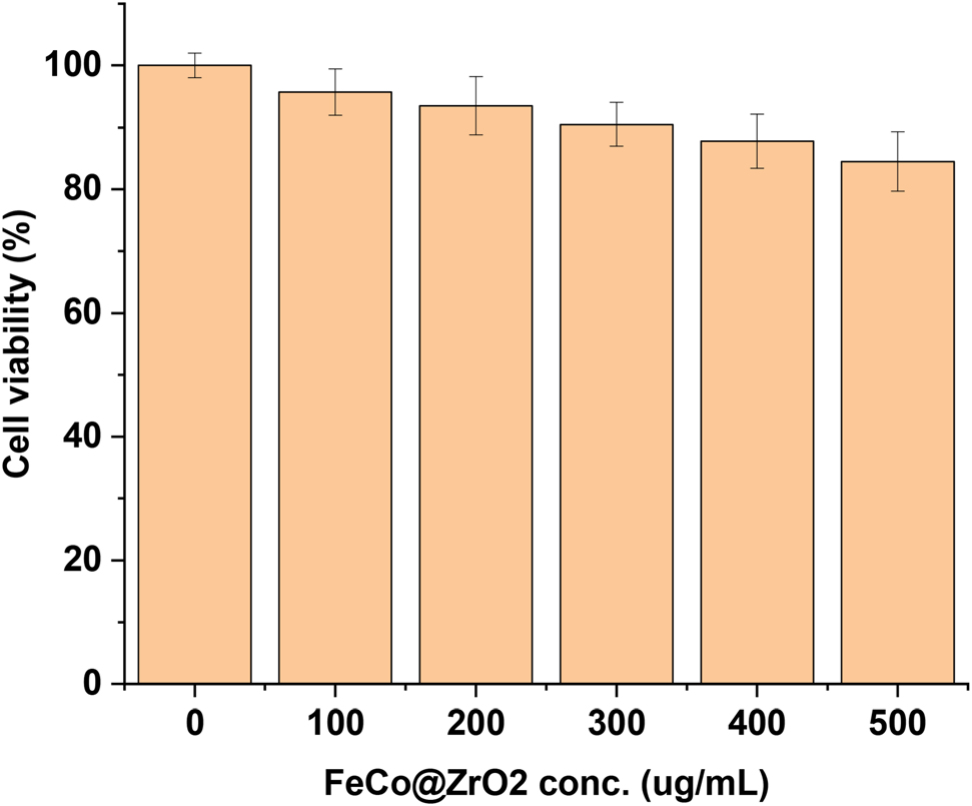
Cytotoxicity of FeCo@ZrO_2_ samples at varying concentrations.

The cytotoxicity of the ZrO_2_-modified FeCo nanoparticles was evaluated using the MTT assay, and the results are presented in Fig. 12. The nanoparticles exhibit good cytocompatibility throughout the investigated concentration range. Even at the highest tested concentration (500 µg mL^−1^), the cell viability remains approximately 84%, indicating relatively low cytotoxicity and suggesting potential suitability for further biomedical-related investigations.

The favorable cytocompatibility may be associated with the high chemical stability, chemical inertness, and low biological reactivity of ZrO_2_ reported in previous studies.^40^ The presence of ZrO_2_ on the nanoparticle surface is expected to reduce direct exposure of the metallic FeCo core to the surrounding biological medium, thereby providing a passivating barrier against undesirable surface reactions. In addition, the hydrophilic nature of zirconia may contribute to improved colloidal stability in aqueous environments, reducing severe particle aggregation and promoting more uniform nanoparticle–cell interactions. Although the specific mechanisms responsible for the observed cytocompatibility were not directly investigated in this work, the relatively high cell viability suggests that the ZrO_2_ surface modification does not introduce significant additional cytotoxic effects.

These results complement the structural and magnetic analyses presented above. While ZrO_2_ modification reduces the saturation magnetization through magnetic dilution effects, the nanoparticles retain substantial magnetic responsiveness together with acceptable cytocompatibility. This balance between magnetic performance and surface stabilization highlights the potential of ZrO_2_-modified FeCo nanoparticles for applications requiring both magnetic functionality and biological compatibility.

Although the ZrO_2_ coating is expected to improve the chemical stability of FeCo nanoparticles and has demonstrated favorable cytocompatibility in the present study, additional investigations are required to evaluate their long-term behavior in physiologically relevant media. In particular, future studies should address colloidal stability, hydrodynamic size evolution, zeta potential, and oxidation resistance under biological conditions. Such studies were beyond the scope of the present work and will be the subject of future investigations. A more comprehensive understanding of these factors will further clarify the role of the ZrO_2_ shell in determining the long-term performance of FeCo nanoparticles for biomedical applications.

## 4 Conclusion

In this work, FeCo nanoparticles were successfully synthesized *via* an ultrasound-assisted co-precipitation method, followed by surface modification using ZrO_2_, PEG, and chitosan through distinct surface functionalization strategies. Optimization of synthesis conditions indicated that an equiatomic Fe : Co ratio combined with moderate thermal treatment at 100 °C yields phase-pure FeCo nanoparticles with the highest saturation magnetization.

Structural analyses using XRD, TEM, SAED, and FTIR confirm the formation of crystalline bcc FeCo nanoparticles with relatively uniform nanoscale morphology after surface modification, while preserving the metallic core structure without detectable phase transformation. The results indicate that surface modification primarily affects the nanoparticle surface rather than the bulk crystalline structure of FeCo.

Magnetic measurements show that pristine FeCo nanoparticles exhibit a high saturation magnetization of approximately 187 emu g^−1^ with low coercivity, consistent with soft ferromagnetic behavior. After surface modification, all samples retain their intrinsic soft magnetic characteristics, although a systematic reduction in saturation magnetization is observed. This reduction is interpreted within a unified framework involving magnetic dilution due to non-magnetic surface species and interfacial spin disorder at the nanoparticle surface, both of which contribute to an effective magnetically inactive region.

Among the investigated surface modifications, ZrO_2_ leads to the most pronounced decrease in saturation magnetization. This behavior is reflected in a larger effective magnetic depletion parameter (*δ*_mag_) compared with polymer-coated samples. The correlation between *M*_s_ and *δ*_mag_ provides a consistent comparative description of how different surface chemistries influence the magnetic response; however, this relationship should be understood as an effective scaling behavior rather than a strict universal law.

Importantly, the ZrO_2_-modified FeCo nanoparticles exhibit good cytocompatibility, with cell viability remaining approximately 84% at 500 µg mL^−1^, indicating that the introduction of an inorganic surface layer does not significantly compromise biological compatibility.

Overall, this study demonstrates that interface engineering provides an effective approach to tailoring the balance between magnetic response and surface-related effects in FeCo nanoparticles. The combination of high intrinsic magnetization, preserved soft magnetic behavior, and tunable surface chemistry highlights the potential of FeCo-based nanoparticles for applications requiring magnetic functionality and surface stability.

## Ethical approval

The authors clarify that this study involved only established, commercially available cell lines (HeLa human cervical cancer cells). These cell lines were obtained from Merck. As no human subjects, primary human tissues, or live vertebrate animals were used in this research, institutional ethical approval was not required according to the Van Lang University, Viet Nam.

## Biosafety statement

All *in vitro* experiments involving human-derived cell lines (HeLa) were conducted in a Biosafety Level 2 (BSL-2) certified laboratory. Standard operating procedures for the handling and disposal of biohazardous materials were strictly followed to ensure researcher and environmental safety.

## Author contributions

Bui The Huy: synthesis samples, methodology, writing – original draft. Jong Won Chung: investigation, formal analysis, validation, writing – review & editing. Tran Thi Ngoc Nha: resources, writing – original draft, Le Thi Tuyet Ngan: data curation, investigation, formal analysis. The-Long Phan and Pham Thanh Phong: conceptualization, methodology, writing – review & editing.

## Conflicts of interest

The authors declare that they have no conflict of interest.

## Data Availability

The data that support the findings of this study are available within the article.
